# Pelvic and Perineal Reconstruction After Bowel, Gynecological or Sacral Tumor Resection: A Case Series

**DOI:** 10.3390/jcm14093172

**Published:** 2025-05-03

**Authors:** Aikaterini Bini, Spyridon Stavrianos

**Affiliations:** Plastic and Reconstructive Surgery Department, Athens General Anticancer-Oncology Hospital “Aghios Savvas”, 11522 Athens, Greece; sstavria@otenet.gr

**Keywords:** perineum, perineal tumors, pelvic reconstruction, rectus abdominis flap, gracilis flap, sacral chordoma

## Abstract

**Background/Aim:** Perineal, pelvic and urogenital reconstruction presents a challenge, not only due to defect size but also due to high morbidity resulting from surgery and post-operative complications. The purpose of this study is to review the surgical approach and evaluate the results regarding pelvic/perineal reconstruction after advanced tumor resection. **Patients and Methods:** The total number of patients was 34 (11 males, 23 females). The histology varied, including sixteen rectal-anal squamous cell carcinomas, five Buschke-Lowenstein tumors, four vulvar-vaginal carcinomas, four sacral chordomas, two cutaneous squamous cell carcinomas, two soft tissue sarcomas and a case of Paget’s disease. Most patients had previously been treated with colectomies and/or gynecological resections and received a full dose of radiotherapy. Reconstruction was performed with the following flaps: oblique/vertical rectus abdominis myocutaneous flap (ORAM/VRAM), gracilis myocutaneous flap, inferior gluteal artery perforator flap (IGAP), internal pudendal artery perforator flap (IPAP) and lotus petal flaps. **Results:** Most patients had a relatively uncomplicated post-operative course. Surgical site infection and wound dehiscence occurred more commonly with the thigh flaps rather than the abdominal flaps. However, the aggression and the frequent recurrences of these tumors had as a result, only 15 out of 34 patients achieved a five-year disease-free survival. **Conclusions:** Pelvic and perineal defects are usually massive and the use of myocutaneous flaps to eliminate the dead space is of paramount importance. Although these are mainly salvage operations with a low survival rate, they promote patients’ quality of life. A frequent challenge is the simultaneous achievement of tumor radical resection and pelvis functionality.

## 1. Introduction

Pelvic and perineal areas are affected by a heterogeneous spectrum of neoplasms with a tendency to recur. The histology varies, including rectal and vaginal squamous cell carcinomas, Buschke-Lowenstein and Bowen disease, as the most common tumors. More rare and aggressive histological types like bone and soft tissue sarcomas can also develop.

Modern management requires a multidisciplinary approach. Surgical resection remains the gold standard. Primary reconstruction may reduce morbidity and shorten recovery. The simultaneous achievement of negative histological margins and restoration of pelvis functionality is usually difficult, while tumor recurrence is considered to be a common phenomenon. Patients frequently present with previous surgical procedures and chemoradiotherapy, seeking further treatment. The majority of them are also elderly and frail, suffering from comorbidities [1].

Extensive perineal defect reconstruction can be challenging owing to numerous factors, including neoadjuvant radiotherapy, closure tension, bacterial contamination of the perineal region and fluid accumulation in the pelvic cavity. Complications of perineal wounds have been reported to be between 25% and 60%. A wide range of reconstructive techniques has been described, including rectus abdominis, gluteus maximus, gracilis, anterolateral thigh and fasciocutaneous lotus petal flaps [2].

The purpose of this study is to review the surgical approach, including reconstruction methods and evaluate the results of adult patients with advanced bowel, gynecological or sacral tumors treated in the Greek Anticancer Institute, a tertiary referral center. Taking into consideration the post-operative complications along with the recurrence rates, the present study aims to demonstrate that surgical resection with negative margins remains the gold standard for the management of pelvic and perineum malignancies, as it can not only improve the quality of life but also prolong the disease-free and the overall survival.

## 2. Patients and Methods

The present study is a retrospective study. All procedures were performed in compliance with relevant laws and institutional guidelines and have been approved by the appropriate institutional committee (reference number of ethical approval: 14825). All patients have given consent for the possible publication of their cases and illustrations. No recognizable features are included in the illustrations. Details regarding personal information and identification remain absolutely anonymous and confidential.

During the study interval (2003–2023), the medical records of 34 consecutive patients admitted for management of advanced perineal and pelvic malignancies were reviewed. The selection criteria included adults with malignancies in the perineal area, including bowel, gynecological or sacral tumors, as well as skin cancer and soft tissue sarcomas. Demographic data were recorded. There were 11 males and 23 females with an average age of 55 years. The anatomical area of the primary tumor, along with the surgical approach and reconstruction technique, were also recorded. Where necessary, the histopathology was reviewed to confirm the diagnosis or derive additional information not included in the original report. Excision margins were histologically complete in all cases.

**Figure 1:**
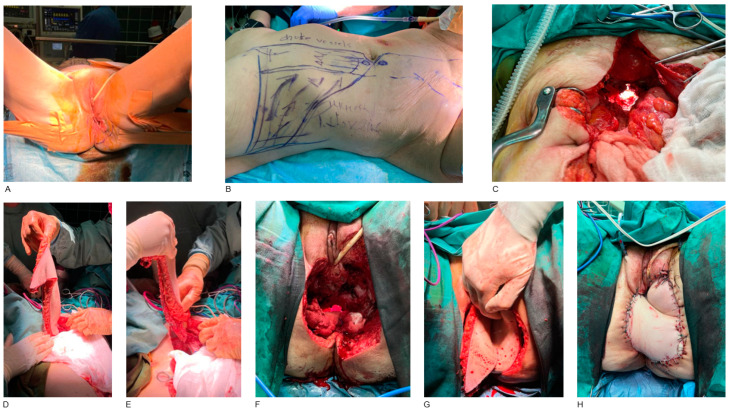
Abdominoperineal resection of perianal SCC and reconstruction with ORAM flap. A 45-year-old female patient with a recurrent perianal SCC extending to the perineum. The patient had previously undergone loop colostomy and received radiotherapy (**A**). Pre-operatively marking of the right ORAM myocutaneous flap and the area of the right deep inferior epigastric vessels (**B**). Abdominal view of the huge volume defect and dead space in the pelvis created after abdominoperineal resection, including sigmoidectomy with end colostomy, proctocolectomy and total hysterectomy (**C**). Harvesting of right ORAM myocutaneous flap (**D**). The whole length of the ORAM flap with its pedicle (**E**). Perineal defect after wide-field surgical resection of the perineum, anus, posterior wall of the vagina and posterior part of labia majora and labia minora (**F**). Transfer of ORAM flap from the abdominal wall, through the pelvis, to the perineal defect (**G**). Post-operative result after perineal reconstruction with ORAM flap (**H**).

The most common anatomical site was the rectum and anus. There were four males and twelve females with such neoplasms. All these patients had already undergone surgeries for loop ileostomy or end colostomy, followed by adjuvant radiotherapy as the primary treatment. They presented after 9 to 12 months post-radiotherapy with recurrent tumors extended to the perineal and pelvic area, having as a result a frozen pelvis. Abdominoperineal resection with pelvic exenteration, including sigmoidectomy, proctocolectomy, and in females also total hysterectomy was performed. In all cases, the histology revealed squamous cell carcinomas. Pelvic reconstruction was performed with VRAM or ORAM flaps based on the defect size (Figure 1). There was also a case of a 47-year-old female with extra-mammary perianal Paget’s disease, where, after tumor resection, the reconstruction was performed with a diamond-shaped perforator flap of the left internal pudendal artery (IPAP).

The second most common anatomical site was the external genitalia. Five females with carcinomas in labia majora and/or labia minora were included. The histology revealed Buschke-Lowenstein tumors in all cases. Reconstruction was performed with unilateral gracilis myocutaneous flap in two cases, bilateral gracilis flap in one case, internal pudendal artery perforator flap (IPAP) in one case and lotus petal flap in one case, respectively. The decision on which reconstruction method to use was based on the defect size and the topographic area of the primary tumor (near the mons pubis or near the perineum).

**Figure 2:**
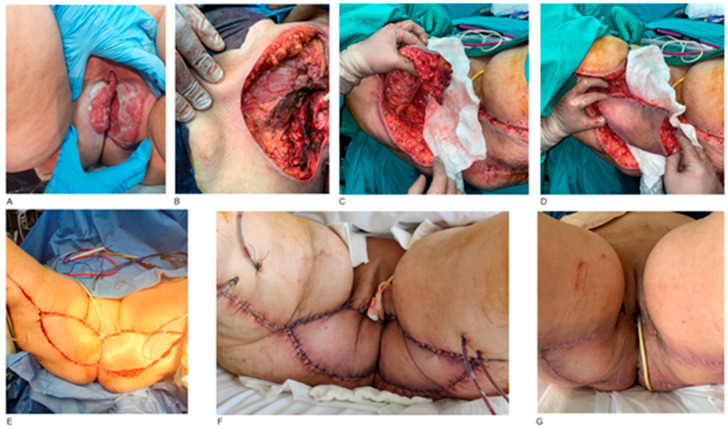
Vulvar-vaginal carcinoma resection and reconstruction with bilateral gracilis flaps. A 52-year-old female patient with an extended vulvar and vaginal squamous cell carcinoma (**A**). The patient underwent wide-field surgical resection of the tumor, including bilateral excision of labia majora, labia minora and vagina (**B**). Harvesting of V-Y bilateral pedicled gracilis myocutaneous flaps (**C**,**D**). The final post-operative result of vulvar-vaginal and perineal reconstruction (**E**). The patient’s hospitalization was uncomplicated and seven days post-operatively, both flaps survived (**F**). Two weeks post-operatively, the flaps had a normal healing process without any signs of infection or wound dehiscence. The patient also had an end colostomy, which minimizes post-operative complications in perineal reconstruction (**G**).

Four females presented with a combination of vulvar-vaginal squamous cell carcinomas. The tumor resection included labia majora, labia minora, perineum and vagina, followed by reconstruction with Y-V bilateral pedicled gracilis myocutaneous flaps. These patients underwent, at the same time, end colostomy in order to minimize post-operative complications (Figure 2).

**Figure 3:**
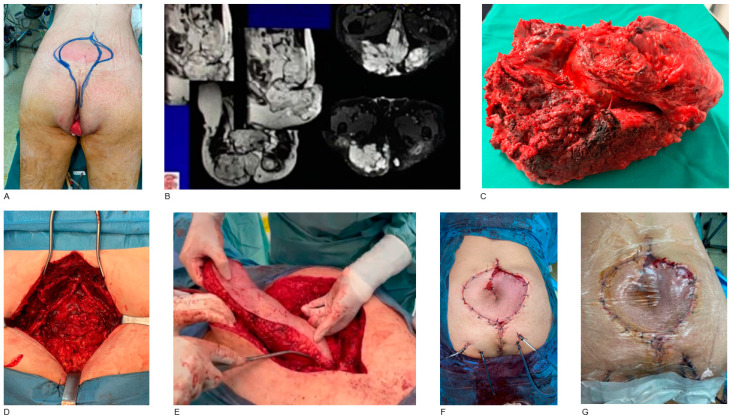
Sacral chordoma resection and sacrum reconstruction with VRAM flap. A 58-year-old male patient with a sacral chordoma. Pre-operative marking of the wide-field resection margins (**A**). T2-weighted and T2-weighted fat sat MR images of the sacrum with intravenous contrast. Sacral chordoma is demonstrated as a hyperintense lesion with irregular margins (**B**). En-block resection of tumor and sacrum (**C**). The patient is in prone position. Tissue and volume defect was created after sacral chordoma wide-field resection. The small intestine is visible, as the defect is connected with the peritoneal cavity (**D**). Transfer of VRAM flap from the abdominal wall, through the peritoneal cavity and pelvis, to the sacral area (**E**). Post-operative result after sacrum reconstruction with transpelvic VRAM flap (**F**). Application of negative pressure wound therapy (NPWT) aiming at a proper wound healing process and avoidance of wound dehiscence (**G**).

Four male individuals with sacral chordomas have also been managed. The tumor origin was the sacrum, which extended to surrounding tissues. Total sacrectomy was performed, followed by reconstruction with transpelvic oblique rectus abdominis myocutaneous (ORAM) flap in three cases and VRAM flap in one case (Figure 3).

**Figure 4:**
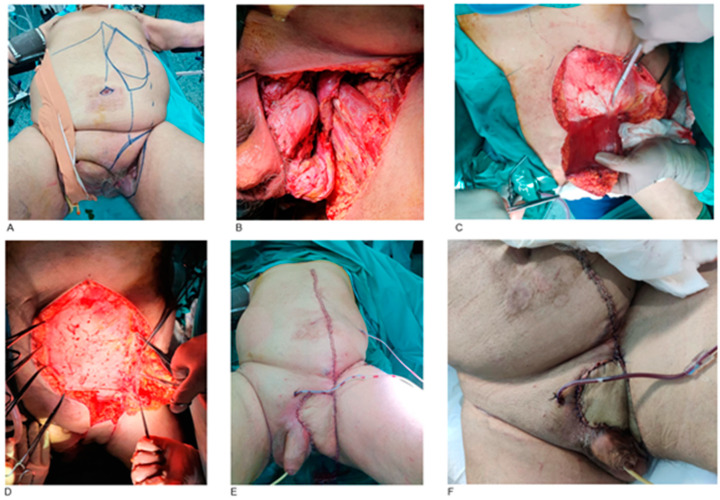
Left inguinal fold SCC resection and reconstruction with VRAM flap. A 67-year-old male patient with a cutaneous SCC in the left inguinal fold. Pre-operative marking of the tumor resection margins, as well as drawing of the left VRAM flap (**A**). Defect after tumor resection with visible left testicle, epididymis and spermatic cord (**B**). Harvesting of left VRAM myocutaneous flap (**C**). Abdominal wall closure in layers, starting from the posterior wall of the rectus abdominis sheath (**D**). Post-operative result of left inguinal fold reconstruction with VRAM flap (**E**). The patient’s hospitalization was uncomplicated and 10 days post-operatively, the flap survived (**F**).

The left inguinal fold was involved in two males presented with cutaneous squamous cell carcinomas. After tumor resection, the spermatic cord and femoral vessels were covered with an ipsilateral VRAM flap (Figure 4).

The gluteal region was another anatomical site involved in two cases: a malignant granular cell tumor (mGCT) and a liposarcoma. The mGCT developed in a 79-year-old male within the left gluteus maximus muscle. The liposarcoma developed in a 67-year-old female in the right gluteal region, above the gluteus muscles. In both cases, tumor resection was followed by reconstruction with an inferior gluteal artery perforator flap (IGAP).

Intraoperatively, a group of different surgical specialists participated, including general surgeons, orthopedic surgeons, urologists, gynecologists and plastic surgeons, aiming at achieving optimal outcomes.

## 3. Results

All patients remained systemically stable during hospitalization. Post-operative surgical complications depended on the anatomical site of the primary tumor as well as the donor site, with thigh flaps presenting more frequent complications than abdominal flaps. In order to purely correlate the complications with the anatomical site of the primary tumor and the flap selection, patient underlying conditions were not analyzed in the present study.

There were four cases of wound dehiscence: two cases of bilateral gracilis myocutaneous flaps for vaginal reconstruction, a case of unilateral internal pudendal artery perforator flap (IPAP) and a case of lotus petal flap for external female genitalia reconstruction. The poor hygiene of the gynecological area, as well as fluid production and moisture may contribute to the impaired healing process and wound breakdown. There were also three cases of wound hematoma that required surgical drainage: two cases of ORAM flap for post-abdominoperineal resection reconstruction and one case of VRAM flap for sacrum reconstruction. Surgical site infection developed in a case of bilateral gracilis myocutaneous flap for vaginal reconstruction, as well as in a case of ORAM flap for sacrum reconstruction; both were treated with intravenous antibiotics for two weeks. Partial venous stasis occurred in the case of an inferior gluteal artery perforator flap (IGAP), which was performed after liposarcoma resection of the gluteal region. Conservative management with sutures release was decided. Partial flap necrosis occurred in a case of ORAM flap for sacrum reconstruction five days post-operatively, where debridement and negative pressure wound therapy (NPWT) application were performed and the wound healed completely after three months. Additionally, there was a case of VRAM flap for inguinal fold reconstruction, where total flap necrosis occurred, leading to re-operation, where a left pedicled anterolateral thigh (ALT) flap was used. Despite these complications, all patients eventually did well and were discharged after 20 to 30 days post-operatively.

The follow-up was every 6 months for a five-year period. Rectum-anus carcinomas presented the worst prognosis with the highest local recurrence rate; only three out of sixteen patients are still alive and disease-free. Sacral chordomas also had a dismal prognosis; three out of four patients developed recurrence and disease progression. Two patients with vulvar-vaginal carcinomas and one patient with external genitalia involvement presented also with recurrent disease. All recurrent tumors were treated with palliative radiotherapy in order to alleviate symptoms and discomfort.

The following cases had a more favorable prognosis. Four patients with Buschke-Lowenstein tumors and two patients with vulvar-vaginal carcinomas achieved a five-year disease-free survival. Inguinal fold carcinomas had a good prognosis without recurrence. The patient with extra-mammary perianal Paget’s disease remains disease-free and has returned to her daily activities. Although the malignant granular cell tumor was low grade, the patient has no signs of recurrence or distant metastasis 2 years after surgery. The liposarcoma was low grade, as well; however, neither local recurrence nor distal metastasis was identified 5 years post-operatively.

The aggression and frequent recurrences of perineal and pelvic tumors resulted in only 15 out of 34 patients achieving a five-year disease-free survival. Patients who developed disease recurrence, although treated with palliative radiotherapy, died during the five-year follow-up period. (Table 1).

## 4. Discussion

The present study evaluated 34 patients who developed pelvic or perineal neoplasms and were surgically managed with wide excisions followed by locoregional flap reconstruction. The incidence, risk factors, anatomical sites, histological subtypes, reconstruction methods, outcomes, complications associated with the surgical interventions, as well as recurrence rates were carefully analyzed in order to provide a comprehensive understanding of the optimal management strategies.

### 4.1. Anatomical Site and Histological Subtypes

- Rectum, anus and perianal area

The most common malignant tumors of the rectum and anus are adenocarcinomas and squamous cell carcinomas, respectively. Anorectal soft tissue tumors are uncommon neoplasms, constituting less than 0.1% of rectal neoplasms. Leiomyosarcomas are the most common, followed by rhabdomyosarcoma. Other extremely rare histological subtypes include angiosarcoma, malignant fibrous histiocytoma, dermatofibrosarcoma protuberans, schwannoma and solitary fibrous tumor [3]. In the present study, there were two cases of soft tissue tumors: a malignant granular cell tumor and a liposarcoma, both developed in the gluteal area.

- Vulva and vagina

Approximately 95% of vulvar malignancies are squamous cell carcinoma. Basal cell carcinoma constitutes 2–3%, while melanoma accounts for approximately 10% of all vulvar neoplasms. Although the vulva is the most common site of extramammary Paget’s disease, it only comprises 1–2% of vulvar malignancies. A variety of other rare malignant tumors includes sarcomas (epithelioid sarcoma, leiomyosarcoma, rhabdomyosarcoma, myxoid sarcoma, liposarcoma, dermatofibrosarcoma protuberans and hemangiosarcoma), adenocarcinomas (mucinous adenocarcinoma, eccrine hidradenocarcinoma), Merkel cell carcinoma, lymphomas and metastases [4]. In this study, there were four cases of vulvar-vaginal squamous cell carcinomas and five cases of Buschke-Lowenstein tumor in the external genitalia.

- Sacrum

Chordoma is a slow-growing and locally aggressive malignant tumor with a predilection for the sacrum. It arises from remnants of the notochord and accounts for 1.4% (range from 1% to 4%) of all primary bone tumors but only just 0.2% of spinal tumors. Despite its rarity, chordoma is the most common primary malignant neoplasm of the sacrum [5,6]. In the present study, there were four cases of sacral chordomas.

### 4.2. Reconstruction Methods

During reconstruction, consideration of both the post-operative appearance of the perineum and the pelvic functionality is essential and must be planned for. Various reconstructive procedures include direct closure, skin grafts, local skin flaps, a variety of myocutaneous and fasciocutaneous flaps and free tissue transfer in rare situations [7].

The choice of the reconstruction method should take into account the wound defect, the patient’s comorbidity, risk of wound complications, existing scars, hernia or stomas, donor site morbidity, as well as the functional and aesthetic result. Factors that could affect the healing process, especially smoking, diabetes or immune suppression, should be identified and recorded pre-operatively. Previous radiotherapy in the wound area is associated with suboptimal wound healing and increases complication risks [8].

- External pelvis and perineum

Defects of the external pelvis and perineal lining are usually amenable to coverage with local or locoregional fasciocutaneous flaps. The use of perforator-based muscle-sparing flaps in suitable instances has increasingly been reported. These flaps depend on the integrity of the vascular territories of the internal pudendal artery, the upper medial thigh plexus, or descending branches of the inferior gluteal artery. The location and extent of the resection usually determine the requirements of the reconstruction and may dictate the choice of the most appropriate flap [1].

- Vulva and vagina

In partial vaginal defects, there is usually sufficient tissue for a local mucosal flap. In small defects, different skin flaps (e.g., Singapore, lotus, gluteal fold, groin flaps, medial thigh flaps) are also versatile. The technique of lowermost lotus petal flaps, designed along the gluteal folds and based on the internal pudendal artery perforators, offers several advantages. The flap vascularity is not compromised by the extensive surgery necessitated in radical vulvectomy and bilateral inguinal dissection. Additionally, the raised flaps are not in the pathway of cancer spread. Furthermore, the donor site scars are excellent, being hidden along the gluteal folds [9].

In case of wound healing compromising factors, like previous radiotherapy or a fistula to adjacent structures (bladder, rectum), filling the defect with well-vascularized muscle tissue (usually gracilis) is considered to be beneficial. Pedicled transverse myocutaneous gracilis flap, either unilateral or bilateral, including both muscle and skin components, is a good choice for vaginal reconstruction. In larger defects (over half of the vagina), abdominal flaps have traditionally been proposed. Deep inferior epigastric artery perforator (DIEP) flap reaches the region on its pedicle and is a good option if the suprapubic approach is performed [10]. In the present study, gracilis myocutaneous flap was performed in all four cases for vulvar-vaginal reconstruction, whereas for external genitalia reconstruction gracilis (*n* = 3), IPAP (*n* = 1) and lotus petal (*n* = 1) flaps were selected.

- Internal pelvis and perineum

When defects involve both the internal pelvis and perineum, myocutaneous flaps are of proven advantage in dealing with both the resurfacing and the bulk needed to fill the pelvic cavity after extensive resections. Rectus abdominis, gluteus and gracilis are the best-known options. Due to the intrinsic limitations of the gracilis flap, rectus and gluteus flaps have largely superseded their role in the majority of cases. The rectus abdominis flap, in particular, provides good bulk and reliable skin and it is the most widely used technique for extensive perineal reconstruction [1]. However, this flap is associated with significant donor site morbidity such as abdominal dehiscence, wound infection, abdominal wall weakening and incisional hernia [2]. On the other hand, the overall complication rate with the gracilis myocutaneous flap is comparable with the more commonly used vertical rectus abdominis myocutaneous flap. Nevertheless, the potential for donor site complications and the severity of those are reduced with thigh flaps. Therefore, gracilis can be considered an acceptable alternative to abdominal flaps for selected perineal defects [11].

The gluteal fold flap is also a reliable, versatile and robust option for perineal reconstruction after extended anorectal resection, despite local radiotherapy, and should be considered for medium and selected large defects [12]. Moreover, no significant difference has been found between muscle-based gracilis flap and perforator-based gluteal fold flap, as both can be reliable and robust techniques for perineal/pelvic reconstruction. Both can effectively obliterate the dead space and even if bilateral myocutaneous gracilis or gluteal fold mobilization is required, morbidity is reduced compared to VRAM flaps [13].

Pedicled anterolateral thigh flap (ALT) may also be used for soft tissue reconstruction of complex defects of the lower abdomen, groin, perineum and hip region. Pedicled perforator-based flaps like ALT minimize functional deficits by preserving muscle. The harvesting technique consists of a retrograde, intramuscular pedicle dissection, which allows sufficient length to be gained in order to transfer the flap into the defect. Tunneling beneath the rectus femoris muscle and sartorius muscle is usually required for tension-free inset [14]. In the present study, pedicled ALT was not selected as a primary-choice flap for perineal reconstruction. However, there was one case where pedicled ALT was performed as a second-choice flap for left inguinal fold reconstruction after total necrosis of the initial VRAM flap.

The fasciocutaneous lotus petal flap based on the internal pudendal artery also remains an appealing option for reconstructing extensive perineal defects following abdominoperineal resection, as it may offer less donor site morbidity and fewer complication rates in comparison to the VRAM flap. Although this technique is simple, fast and straightforward, it remains limited due to its size, with the mean size of this flap reported to be around 14 cm in length and 6 cm in width [2]. Propeller flaps, transposition flaps and V-Y advancement flaps based on internal pudendal artery perforators with reliable blood circulation can offer suitable volume not only for vulvar-vaginal and anal reconstruction, which requires thin flaps, but also for pelvic floor reconstruction, which requires flap volume [15]. In this study, two internal pudendal artery perforator (IPAP) flaps and a lotus petal flap were performed to cover defects in the perianal area and the external genitalia. There were no cases of IPAP flaps for large pelvic/perineal defects after abdominoperineal resection, as the tissue and volume loss could only be covered with bulky ORAM/VRAM flaps.

- Sacrum

After partial or total sacrectomy for chordoma excision, a wide variety of flaps are available based on the defect size. Local gluteal flaps offer the advantage of low donor site morbidity and they are close to the same surgical field. They are usually suitable to cover small to medium defects and sometimes larger ones if there is excess adipose tissue in the gluteal region. The disadvantage is sometimes the venous congestion of the gluteal flap. On the other hand, the VRAM flap is selected after total sacrectomies in case of larger defects and mostly in patients without previous abdominal surgery. Three main disadvantages of the VRAM flap technique are the risk of abdominal donor side hernia, the pedicle compression inside the transpelvic tunnel, which may compromise the flap blood flow, and the patient transfer from lithotomy to a prone position, increasing the overall surgical time [5]. In the present study, the tissue defect and volume loss were excessive after sacrectomies and therefore transpelvic VRAM/ORAM flap was performed in all four cases after chordoma resection. A free flap could be an alternative option when local gluteal flaps or transpelvic VRAM cannot be performed due to previous radiotherapy or abdominal surgery. However, the dissection of gluteal vessel branches is as tedious and complicated as that of the recipient vessels.

- Free flaps

The role of free tissue transfer for perineal reconstruction remains limited to isolated cases that are not suitable for the current standard techniques [1]. Well-vascularized free flap coverage is usually required for massive and extensive defects of the perineal or inguinal area. Perforators arising from superficial circumflex iliac vessels, superficial inferior epigastric vessels, superficial and deep external pudendal vessels, lateral and medial circumflex femoral vessels and internal pudendal vessels yield acceptable flap survival [16]. In the present study, there were no cases of free flap reconstruction.

- Biological meshes

Abdominoperineal resection may have significant complications, especially when requiring pelvic reconstruction with myocutaneous flaps. Biological meshes are a novel alternative and have been reported to be a safe approach for perineum reconstruction [17]. The available data regarding the optimal technique are still limited. However, the existing literature suggests that there is no significant difference in complication rates between biological meshes and flap reconstruction [18].

## 5. Conclusions

Major anorectal and gynecological cancer surgery involving the pelvis and perineum can cause large soft tissue defects that require reconstruction with plastic surgery techniques. Currently, several alternatives are available for the reconstruction of the perineum.

Although these are mainly salvage operations with a low survival rate, the results of this study have confirmed that they promote patients’ quality of life when performed in tertiary referral centers by surgeons with many years of experience in managing complex oncological cases.

New and innovative reconstructive modifications keep appearing and larger case series are needed for more evidence-based information on the outcomes achieved.

## Figures and Tables

**Table 1:** The anatomical site, gender, histopathology of perineal/pelvic malignancies and reconstruction methods.

Anatomical Site and Gender	Histopathology	Reconstruction	Recurrence
Rectum-anus (M = 4, F = 12)	SCC (*n* = 16)	ORAM/VRAM (*n* = 16)	*n* = 13
Perianal region (F = 1)	EMPD (*n* = 1)	IPAP (*n* = 1)	*n* = 0
External genitalia (F = 5)	Buschke-Lowenstein (*n* = 5)	Gracilis (*n* = 3) IPAP (*n* = 1) Lotus petal (*n* = 1)	*n* = 1
Vulva-Vagina (F = 4)	SCC (*n* = 4)	Gracilis (*n* = 4)	*n* = 2
Sacrum (M = 4)	Chordomas (*n* = 4)	ORAM/VRAM (*n* = 4)	*n* = 3
Inguinal fold (M = 2)	SCC (*n* = 2)	VRAM (*n* = 2)	*n* = 0
Gluteal region (M = 1, F = 1)	mGCT (*n* = 1, male)	IGAP (*n* = 1)	*n* = 0
	Liposarcoma (*n* = 1, female)	IGAP (*n* = 1)	*n* = 0

M: male, F: female, SCC: squamous cell carcinoma, ORAM/VRAM: oblique/vertical rectus abdominis myocutaneous flap, EMPD: extra-mammary perianal Paget’s disease, IPAP: internal pudendal artery perforator flap, IGAP: inferior gluteal artery perforator flap, mGCT: malignant granular cell tumor.

## Data Availability

The data presented in this study may be available on request from the corresponding author due to privacy and patient confidentiality.
